# Intensive care nurses’ knowledge and practice of evidence-based recommendations for endotracheal suctioning: a multisite cross-sectional study in Changsha, China

**DOI:** 10.1186/s12912-021-00715-y

**Published:** 2021-10-04

**Authors:** Wenjun Chen, Shuang Hu, Xiaoli Liu, Nina Wang, Junqiang Zhao, Peng Liu, Kaixia Chen, Jiale Hu

**Affiliations:** 1 School of Nursing, University of Ottawa, 451 Smyth Road, Ontario Ottawa, Canada; 2 Center for Research on Health and Nursing, University of Ottawa, Ottawa, Ontario Canada; 3 School of Nursing, Changsha Medical University, Hunan Changsha, People’s Republic of China; 4 Operating Room, Peking University People’s Hospital, Beijing, People’s Republic of China; 5 Department of Respiratory Medicine, Xiangya Hospital, Central South University, Changsha, People’s Republic of China; 6 Cardiovascular Surgery ICU, Xiangya Hospital, Central South University, Changsha, People’s Republic of China; 7 Paediatric Unit, Meitan Chinese and Western Integrative Medicine Hospital, Zunyi, People’s Republic of China; 8 Department of Nurse Anesthesia, Virginia Commonwealth University, VA Richmond, USA

**Keywords:** Intensive care units, Suction, Evidence-based practice, Nursing, China

## Abstract

**Background:**

Endotracheal suctioning is one of the most frequently performed invasive procedures by intensive care nurses. Nurses should have adequate knowledge and skills to perform endotracheal suctioning based on the best evidence. Little is known about intensive care nurses’ knowledge and practice of evidence-based endotracheal suctioning in Chinese hospitals. The purpose of this study was to investigate intensive care nurses’ knowledge and practice of evidence-based recommendations regarding endotracheal suctioning. Specifically, the study aimed to examine (1) intensive care nurses’ awareness of and adherence to endotracheal suctioning guidelines and (2) factors influencing their level of awareness and adherence.

**Methods:**

A cross-sectional survey of 310 staff nurses working in intensive care units was carried out at Changsha, China. Data on participants’ characteristics, awareness of, and adherence to the endotracheal suctioning guidelines were collected through online questionnaires. Following univariate descriptive statistics, the Mann–Whitney U test and Kruskal–Wallis H test were performed using Software Package Statistical Analysis Version 23.0.

**Results:**

A total of 281 nurses completed and returned the survey (response rate = 90.6 %). One-half to three-quarters of the nurses knew 21 of the 26 evidence-based practices and believed their practices followed the guidelines. Over half of them were unaware of the difference between open and close suctions and the pros and cons of using hyperinflation. Almost 50 % of nurses believed some of their clinical practices did not follow the evidence-based recommendations, such as not routinely using normal saline and using 80–120 mmHg suction pressure during endotracheal suctioning. Nurses with endotracheal suctioning training demonstrated significantly higher awareness of endotracheal suctioning recommendations and higher adherence levels than untrained nurses.

**Conclusions:**

The study findings revealed that Chinese intensive care nurses lacked awareness of several essential evidence-based endotracheal suctioning practices, and there were gaps between their current practice and the guideline recommendations. Further research should emphasize revealing barriers and facilitators of implementing evidence-based endotracheal suctioning practices as well as developing context-suitable interventions for guideline implementation.

## Background

Endotracheal suctioning (ETS) is a component of bronchial hygiene therapy and mechanical ventilation and involves the mechanical aspiration of pulmonary secretions from a patient with an artificial airway in place [1, 2]. The purpose of this procedure is to maintain the patient airway, to optimize ventilation and oxygenation and to prevent respiratory tract infection from the lodgement of secretions [3]. ETS is among the most frequently conducted invasive procedures in the intensive care unit (ICU) for mechanically ventilated patients [4]. As a crucial procedure, if ETS is not performed with correct techniques, it will lead to numerous adverse effects, such as tracheobronchial oedema, ulceration, and denudation of the epithelium [5–7]. These areas of mucosal damage increase the risk of infection and bleeding [8]. Moreover, ETS is considered an extremely distressing and painful experience for ICU patients [9]. Study findings showed that ETS performance by well-educated health care professionals based on the best evidence can diminish its side effects [10, 11]. It is, therefore, essential for health care professionals to have updated knowledge on the evidence-based practices of ETS so that they can perform the procedures scientifically and thereby reduce patients’ complications and potential risks [12].

Clinical practice guidelines are “systematically developed statements to assist practitioner and patient decisions about appropriate health care for specific clinical circumstances” [13]. As a type of high-level evidence in the evidence hierarchy, guidelines provide clinicians trustworthy recommendations and can be used to reduce inappropriate practice variations and promote the delivery of high-quality care [8, 13]. Several ETS guidelines have been developed in recent years by authoritative organizations. For example, in 2010, the American Association of Respiratory Care (AARC) released the AARC Clinical Practice Guidelines on ETS of mechanically ventilated patients with artificial airways [1]. Despite the enormous efforts made in developing those guidelines, there continues to be large discrepancies between evidence and the actual practice on ETS. Researchers in countries such as Canada [8], France [14], Australia [15], and Italy [4] analysed the application of guidelines for endotracheal suctioning among intensive care nurses. Their studies revealed that nurses were often not aware of the existence of those guidelines, and there was a lack of relevant knowledge regarding ETS, leading to ETS practices that were inconsistent with evidence-based recommendations.

In China, intensive care nurses are responsible for performing ETS in ICUs. According to the “Guidelines for the Construction and Management of Critical Care Medicine in China,” the ratio of intensive care nurses to ICU beds should be 2.5–3:1 or greater [16]. However, the ICU nurse-to-bed ratios decreased from 1.8:1 in 2002 to 1.5:1 in 2018, indicating an unsolved severe shortage of ICU nurses in China [17]. Facing such a shortage, in addition to expanding the number of nursing staff, it is critical to understand nursing practice in Chinese ICUs and ensure that nurses are equipped with updated knowledge and skills in performing intensive care practices to ensure patient safety and positive patient outcomes. As one of the most frequently performed procedures in the ICU, nurses’ knowledge and practice of ETS in China have been examined by only a few studies. Gu et al. [18] and Qin et al. [19] found that intensive care nurses had adequate knowledge about the purpose of preoxygenation and humidification, while few were aware of the suction pressure, indications and adverse effects of suction. Gu [18] also revealed that most nurses’ ETS knowledge came from work experience instead of scientific journal research, indicating a lack of knowledge about up-to-date suctioning recommendations among nurses. Zhang et al. [20] evaluated nurses’ compliance with the aseptic technique during ETS by observation and found that nurses frequently forgot to wash their hands before and after performing ETS. Those studies were limited by the small number of survey items and only covered a few aspects of ETS practices, such as catheter size, insertion depth, humidification, aseptic technique suction pressure, and adverse effects [18–20]. Other essential aspects were not included, for example, the thorough assessment of the patient before suctioning to establish the needs and monitoring indicators such as breath sounds, oxygen saturation, and the respiratory rate and pattern throughout the suctioning procedure [21]. Furthermore, all the abovementioned studies were conducted at least 10 years ago, and ETS practice has evolved during the past decade. It is therefore necessary to conduct a comprehensive, current investigation into intensive care nurses’ knowledge of ETS and the practices they perform to establish a robust evidence base for both policy-making and intensive care nurse training in China.

In 2018, Hu et al. [21] developed an adapted guideline on ETS for adult patients with artificial airways. With the guidance of the ADAPTE framework, the guideline was developed through literature review, expert panel consultation (i.e., nurses, physicians and patients, *n* = 9), and external reviewer meeting (*n* = 20) [22]. The final guideline includes 26 recommendations covering 3 procedure phases and 17 points of care. This study aims to examine (1) intensive care nurses’ awareness of and adherence to ETS guidelines and (2) factors influencing nurses’ awareness and practice adherence.

## Method

### Study design

This was a cross-sectional study, and an online questionnaire survey was employed.

### Setting and sampling

Convenient sampling was applied to recruit intensive care nurses from 18 adult ICUs in five tertiary general hospitals in Changsha, the second-most populous city in the central southern part of China. The types of ICUs included general surgery ICUs, cardiothoracic surgery ICUs, neurological ICUs, respiratory ICUs, emergency department ICUs, coronary care units, etc. We included registered nurses who worked in these ICUs and provided direct care for adult patients with artificial airways at the time of investigation, excluding (1) nurses who worked at paediatric ICUs and (2) five nurses who participated in the pre-test.

### Questionnaire survey

We formulated a questionnaire based on recommendations from the adapted endotracheal suctioning (ETS) guidelines after receiving permission from the first author [21]. The guideline contained 26 key recommendations in three procedural phases and 17 points of care [21]. The questionnaire involved three domains: (1) demographic data and (2) nurses’ awareness of and (3) nurses’ adherence to the ETS guidelines. Demographic characteristics included nine items: age, gender, highest level or degree of education, length of nursing employment and ICU experience, type of ICU, job title, number of patients per nurse on duty, and training experience. The second domain included 26 items that were directly extracted from the ETS guidelines. We organized the items as (1) practice prior to suctioning (*n* = 5), such as clinical indicators, patient communication, catheter size, knowledge, and skills; (2) ETS procedure (*n* = 18), including the ETS approach, aseptic technique, humidification, insertion depth, suction pressure, time length and frequency of ETS, suction intervals, hyperinflation, preoxygenation, ventilation; and (3) evaluation after ETS (*n* = 2), including monitoring and adverse effects. The third domain included 16 items, which were also organized as being prior (*n* = 3), during (*n* = 12), and after suctioning (*n* = 1). Two self-rating scales were applied to rate the second and third domains: (1) Are you aware of the recommendation (Yes/No)? (2) Does your current ETS practice adhere to the recommendation (Yes/No)? We aimed to explore the knowledge and practice of intensive care nurses by investigating their awareness of ETS guidelines and perceptions of practice adherence using a questionnaire survey.

The questionnaire was reviewed by five ICU nurse managers who had over ten years of clinical intensive care experience. They provided suggestions and feedback on the wording accuracy and understandability of these items. Based on the review, we revised the wording of four items in the second domain and six items in the third domain for clarity. No items were added or removed. To determine the test-retest reliability of the revised questionnaire, we invited five intensive care nurses to fill out the questionnaire twice with an interval of two weeks, and the correlation coefficient of scores of two tests was calculated by 0.89. Cronbach’s α values for the awareness and adherence scales were 0.835 and 0.812, respectively.

### Data collection

To maintain the anonymity of participants, we disseminated the electronic survey through a WeChat group that was set up specifically for this study and could accommodate up to 500 participants. WeChat is the most commonly used social networking application in China and has been widely used in health care studies for professional education, health care intervention, and questionnaire collection [23]. After obtaining the required authorization from the nursing department and ICU nurse managers of the five hospitals for distributing the questionnaire, participants were recruited by research assistants (RAs) under the supervision of the first author. The RAs were nurses working in those participating hospitals. They disseminated the recruitment information and informed consent forms to the WeChat groups of the ICU nursing teams in the five hospitals. Those intensive care nurses who agreed to participate were added to the WeChat group specifically for data collection. After giving their informed consent, 310 nurses from the five tertiary hospitals were invited to join the WeChat group. Then, a study invitation with a hyperlink to the online survey was sent to the WeChat group. The participants could choose to use a mobile device or desktop computer to complete the survey. Each IP address only allowed participants to complete the survey once. Data were collected automatically upon submission. No personal data or information on the participants was collected.

### Ethical issues

 Research Ethics Committee approval was obtained at the Xiangya Nursing School of Central South University. Before data collection, the aim of the study was explained to the nurses by research assistants with informed consent from the participating hospitals. The questionnaires were nameless, noncoded, and confidential. Participant response bias and any possible enforcement participation were diminished since there was no way to identify the participants.

### Data analysis

Software Package Statistical Analysis (SPSS) Version 23.0 was applied for data analysis [24]. Demographic data were analysed using descriptive statistics. They were also used as independent variables to understand the influencing factors for awareness of and adherence to ETS guidelines. We applied descriptive statistics methods to record numbers and percentage of participants answering Yes/No to each item and in total for the two scales. For statistical inference, nonparametric test methods such as the Mann–Whitney U test and Kruskal–Wallis H test were used to identify the significance in the observed differences in awareness and adherence of ETS scores by independent variables such as gender, years of working in the ICU, and ETS training, considering statistical significance at a *p* value less than 0.05.

## Results

### Demographic characteristics

The survey was conducted from July 2018 to August 2018. Of the 310 intensive care nurses who joined the study’s WeChat groups, 281 completed and returned the questionnaire (response rate = 90.6 %). As shown in Table 1 and 85.77 % (*n* = 241) of the intensive care nurses were female nurses, and one in three (*n* = 186) were aged 20 to 30 years. Over three-quarters of them (*n* = 234) had a bachelor’s degree, and only one had a Ph.D. The majority of the nurses had less than ten years of work experience in the ICU (*n* = 238). Over half of them (*n* = 154) had senior nurse job titles. A total of 60.14 % (*n* = 169) of nurses worked in a general ICU, 16.73 % (*n* = 47) in a respiratory ICU, and only three in a cardiothoracic ICU. A total of 70.46 % (*n* = 198) took care of three to four patients in their practices; 68.68 % (*n* = 193) had received ETS training before.

**Table 1:** Demographic characteristics of intensive care nurses

Characteristic	Group	Frequency	Percentage (%)
Gender	Male	40	14.23
	Female	241	85.77
Age	≤ 20	3	1.07
	21–30	186	66.19
	31–40	80	28.47
	41–50	12	4.27
Highest Level of Education	Associate degree	26	10.94
	Bachelor’s Degree	234	77.10
	Master’s Degree	20	10.94
	PhD	1	0.29
Years of work as registered nurses	≤ 5	110	39.15
	6–10	109	38.79
	11–15	37	13.17
	16–20	13	4.63
	> 20	12	4.27
Years of work in ICU	1–5	135	48.04
	6–10	103	36.65
	11–15	27	9.61
	16–20	10	3.56
	> 20	6	2.14
Type of ICU	General	169	60.14
	Cardiothoracic	3	1.07
	Neurological	8	2.85
	Respiratory	47	16.73
	Emergency (department)	6	2.14
	Coronary	20	7.12
	Others	28	9.96
Job Title	Nurse	40	14.24
	Senior Nurse	154	54.8
	Supervisor Nurse	81	28.83
	Co-Chief Nurse	5	1.78
	Chief Nurse	1	0.36
Number of Patients Per Nurse on Duty	1	6	2.14
	2	47	16.73
	3	101	35.94
	4	97	34.52
	≥ 5	30	10.68
Received specific ETS training	Yes	193	68.68
	No	88	31.32

### Participants’ awareness of endotracheal suctioning

Only five nurses were aware of all the recommendations. Participating nurses were aware of an average of 16 of the 26 recommendations. According to Table 2, only 95 (33.8 %) and 124 (44.1 %) of the nurses knew that “the closed or open suction system is not superior to the other in terms of oxygen saturation, cardiovascular instability, secretion removal, environmental contamination, and cost” (Item 7) and that “tidal volumes should be no more than 900 cc during hyperinflation because patients may feel dyspnoeic” (Item 18), respectively (Table 2)

**Table 2:** Intensive care nurses’ awareness of the ETS guidelines

Practices prior to, during, and post ETS event		Items^a^ (*n* = 26)	Variables	Awareness No. of nurses rate yes/no (%)
Preparation before Endotracheal suctioning	Clinical indicators	1. Suctioning should only be done when a thorough assessment of the patient establishes the need for such a procedure and not be dictated by routine	Yes^**b**^	191 (68.0)
			No	90 (32.0)
	Patient communication	2. If patients are able to cough up their own secretions, they should be encouraged to do so	Yes	192 (68.3)
			No	89 (31.7)
	Catheter size	3. Suction catheters should be as small as possible, yet large enough to facilitate secretion removal	Yes	125 (44.5)
			No	156 (55.5)
		4. The size of the suction catheter should occlude no more than half of the internal diameter of the artificial airway to avoid greater negative pressures in the airway and to potentially minimize falls in PaO2	Yes	175 (62.3)
			No	106 (37.7)
	Knowledge and Skills	5. I possess required procedural skill and gentleness when suctioning because of the potential associated hazards	Yes	193 (68.7)
			No	88 (31.3)
The procedure of Endotracheal suctioning	Suction Approach	6. The use of a closed suction system is suggested for adults with high FIO2 or PEEP, or at risk for acute lung injury	Yes	170 (60.5)
			No	111 (39.5)
		7. The closed or open suction system is not superior to the other in terms of oxygen saturation, cardiovascular instability, secretion removal, environmental contamination, and cost	Yes	95 (33.8)
			No	186 (66.2)
	Aseptic Technique	8. Aseptic technique should be considered an essential component of the suctioning procedure for hospitalized patients with artificial airways, including handwashing and use of gloves because endotracheal suctioning is an invasive procedure that may lead to contamination of the lower airways	Yes	192 (68.3)
			No	89 (31.7)
	Humidification	9. Routine use of normal saline instillation prior to endotracheal suction should not be performed	Yes	164 (58.4)
			No	117 (41.6)
		10. Ensuring patients are adequately hydrated is the way health care providers can facilitate the removal of respiratory secretions	Yes	189 (67.3)
			No	92 (32.7)
	Insertion Depth	11. The suction catheter should be inserted to the carina and then retracted 1–2 cm before suctioning is performed, or the length of the suction catheter is estimated by measuring an identical endotracheal tube	Yes	169 (60.1)
			No	112 (39.9)
		12. Deep suctioning is necessary for patients with large amounts of secretions in the lower airways	Yes	186 (66.2)
			No	95 (33.8)
	Suction Pressure	13. Using the lowest possible suction pressure during endotracheal suctioning, usually 80–120 mmHg	Yes	152 (54.1)
			No	129 (45.9)
	Time Length of Suction Procedure	14. The suctioning procedure should last no longer than 15 s	Yes	193 (68.7)
			No	88 (31.3)
	Frequency of Suction Procedure	15. There should not be more than two consecutive suction procedures	Yes	171 (60.9)
			No	110 (39.1)
	Suction Intervals	16. Perform suctioning at least every 8-hour to reduce the risk of partial occlusion of the endotracheal tube and the accumulation of secretions	Yes	139 (49.5)
			No	142 (50.5)
	Hyperinflation	17. Using volumes of hyperinflation that is indexed to the size of the patient may assist in minimizing potential difficulties	Yes	160 (56.9)
			No	121 (43.1)
		18. Tidal volumes should no more than 900 cc during hyperinflation because patients may feel dyspneic	Yes	124 (44.1)
			No	157 (55.9)
		19. If hyperinflation is used in the patients before suctioning, caution should be employed because it may be associated with increases in mean arterial blood pressure	Yes	138 (49.1)
			No	143 (50.9)
	Pre-oxygenation	20. Pre-oxygenation by the delivery of 100 % oxygen for at least 30 s prior to and after the suctioning procedure is recommended to prevent a decrease in oxygen saturation, especially when the patient has a clinically important reduction in oxygen saturation with suctioning	Yes	188 (66.9)
			No	93 (33.1)
		21. Combining hyperoxygenation and hyperinflation prior to suctioning can minimize suctioning-induced hypoxemia	Yes	143 (50.9)
			No	138 (49.1)
	Ventilation	22. A ventilator should be used rather than a manual resuscitation bag to provide hyperventilation/hyperoxygenation prior to suctioning to reduce hemodynamic alterations	Yes	151 (53.7)
			No	130 (46.3)
		23. Suctioning through an adaptor is preferred to preserve oxygenation in mechanically ventilated patients	Yes	164 (58.4)
			No	117 (41.6)
		24. A washout time of up to two minutes can be required when hyperoxygenation is being delivered via some ventilators to allow time for the increased oxygen percentage to come through the ventilator tubing and reach the patient	Yes	183 (65.1)
			No	98 (34.9)
Evaluation after Endotracheal suctioning	Monitoring	25. The following should be monitored prior to, during, and after the procedure, if indicated and available: breath sounds, oxygen saturation, respiratory rate and pattern, hemodynamic parameters, sputum characteristics, cough characteristics, intracranial pressure, and ventilator parameters	Yes	187 (66.5)
			No	94 (33.5)
	Adverse Effects	26. Endotracheal suctioning, unless managed appropriately, can lead to various adverse events (tracheal trauma, hypoxemia, hypertension, cardiac arrhythmias, and raised intracranial pressure) and increase mortality and morbidity rates	Yes	186 (66.2)
			No	95 (33.8)

^a^All items (*n* = 26) are guideline recommendations [21]

^b^‘Yes’ indicated participants were aware of the recommendation

### Perceived practice adherence to ETS recommendations

Only nine nurses perceived their practice adherence to all 16 ETS recommended practices. Participating nurses were aware of an average of 9 of the 16 practices. As shown in Table 3, items with the least adherence included “use 80–120 mmHg suction pressure during endotracheal suctioning” (Item 9, 145 (51.6 %)) and “do not perform normal saline instillation routinely before endotracheal suction” (Item 6, 141 (50.2 %)).

**Table 3:** Adherence of intensive care nurses’ practice to the ETS guidelines

Practices prior to, during, and post ETS event		Items^a^ (*n* = 16)	Variables	Adherence No. of nurses rate yes/no (%)
Preparation before Endotracheal suctioning	Clinical indicators	1. I assessed the need for endotracheal suctioning as a routine part of the patient/ventilator system assessment	Yes^b^	187 (66.5)
			No	94 (33.5)
	Patient communication	2. I encouraged patients to cough up their own secretions if they are able to	Yes	192 (68.3)
			No	89 (31.7)
	Catheter size	3. I used the suction catheter occlude less than half of the internal diameter of the artificial airway	Yes	167 (59.4)
			No	114 (40.6)
The procedure of Endotracheal suctioning	Suction Approach	4. I used a closed suction system suggested for adults with high FIO2, or PEEP, or at risk for acute lung injury	Yes	156 (55.5)
			No	125 (44.5)
	Aseptic Technique	5. I washed my hands before and after suctioning and wore gloves during suctioning	Yes	190 (67.6)
			No	91 (32.4)
	Humidification	6. I did not perform normal saline instillation routinely before endotracheal suction	Yes	141 (50.2)
			No	140 (49.8)
	Insertion Depth	7. I inserted the suction catheter to the carina and then retracted 1–2 cm before suctioning or measured an identical endotracheal tube to estimate the length of the suction catheter.	Yes	158 (56.2)
			No	123 (43.8)
		8. I performed deep suctioning for patients with large amounts of secretions in the lower airways	Yes	180 (64.1)
			No	101 (35.9)
	Suction Pressure	9. I used 80-120mmHg suction pressure during endotracheal suctioning	Yes	145 (51.6)
			No	136 (48.4)
	Time Length of Suction Procedure	10. I performed each suctioning procedure less than 15 s	Yes	191 (68.0)
			No	90 (32.0)
	Frequency of Suction Procedure	11. I performed less than three consecutive suction procedures each time	Yes	175 (62.3)
			No	106 (37.7)
	Suction Intervals	12. I performed suctioning for each patient at least every 8-hour	Yes	146 (52.0)
			No	135 (48.0)
	Pre-oxygenation	13. I performed pre-oxygenation by delivering 100 % oxygen for at least 30 s prior to and after the suctioning procedure.	Yes	184 (65.5)
			No	97 (34.5)
	Ventilation	14. I used a ventilator instead of a manual resuscitation bag to provide hyperventilation/hyperoxygenation prior to suctioning to reduce hemodynamic alterations	Yes	143 (50.9)
			No	138 (49.1)
		15. I performed suctioning through an adaptor to preserve oxygenation in mechanically ventilated patients	Yes	155 (55.2)
			No	126 (44.8)
Evaluation after Endotracheal suctioning	Monitoring	16. I monitored the following prior to, during, and after the procedure if indicated and available: breath sounds, oxygen saturation, respiratory rate and pattern, hemodynamic parameters, sputum characteristics, cough characteristics, intracranial pressure, and ventilator parameters.	Yes	171 (60.8)
			No	110 (39.2)

^a^All items (*n* = 16) are recommended practices by the guideline [21]

^b^‘Yes’ indicated participants’ practice adherence to the recommendations

### Factors associated with intensive care nurses’ awareness of and adherence to the guidelines

As shown in Table 4, nurses with ETS training experience demonstrated significantly higher awareness of ETS recommendations (*p* = 0.000) and perceived their practices as being more adherent to the recommendations than untrained nurses did (*P* = 0.005).

**Table 4:** Factors associated with nurses’ awareness of and adherence to the guideline

Factors	Variables	No. of nurses answered	Awareness scale			Adherence scale		
			Median (IQR)^a^	Z ^b^ or X^2 c^	*p*-value	Median (IQR)	Z ^b^ or X^2 c^	*p*-value
Gender	Male	40	17(16,18)	Z=-0.139	0.889	11(8,11)	Z=-0.781	0.435
	Female	241	18(15,19)			10(9,11)		
Age	≤20	3	19(n/a)	X^2^=1.817	0.611	11(n/a)	X^2^=0.077	0.994
	21-30	186	17(15-19)			10(9,11)		
	31-40	80	17.5(16,19)			10(8,11)		
	41-50	12	18(16,18.25)			10(9.5,11)		
Highest Level of Education	Associate degree	26	18(16,19)	X^2^=1.764	0.623	11(8,11)	X^2^=3.54	0.316
	Bachelor's Degree	234	17(15,19)			10(9,11)		
	Master's Degree	20	17.5(15,18)			10(7.5,10.5)		
	PhD	1	n/a			n/a		
Years of work as registered nurses	≤5	110	17(15,19)	X^2^=1.439	0.837	10(8,11)	X^2^=2.129	0.712
	6-10	109	17(16,18)			10(9,11)		
	11-15	37	18(15.5,19)			11(8,11)		
	16-20	13	18(16,19)			10(9,11)		
	>20	12	18(15.5,19)			10.5(9,11)		
Years of work in ICU	1-5	135	17(14,19)	X^2^=4.842	0.304	10(8,11)	X^2^=5.021	0.285
	6-10	103	18(15.5,18.5)			10(9,11)		
	11-15	27	18(17,19)			11(9,11)		
	16-20	10	17.5(10.75,19)			10(6,11)		
	>20	6	18(15,19)			11(7,11)		
Type of ICU	General	169	18(16,19)	X^2^=9.312	0.071	10(9,11)	X^2^=8.916	0.078
	Cardiothoracic	3	17(n/a)			10(n/a)		
	Neurological	8	14(10.5,16)			7.5(6,9)		
	Respiratory	47	17(15,19)			11(9,11)		
	Emergency (department)	6	17(10.75,19)			9.5(6,11)		
	Coronary	20	19(17.25,19)			11(10,11)		
	Others	28	17(13.5,19)			10(6,11)		
Job Title	Nurse	40	17(14.25,19)	X^2^=3.302	0.509	9.5(8,11)	X^2^=8.183	0.085
	Senior Nurse	154	17(15,19)			10(9,11)		
	Supervisor Nurse	81	18(16,19)			10(9,11)		
	Co-Chief Nurse	5	18(17,18)			10(9,10)		
	Chief Nurse	1	n/a			n/a		
Number of Patients Per Nurse on Duty	1	6	17.5(15,19)	X^2^=1.896	0.755	10(9,11)	X^2^=4.059	0.398
	2	47	18(16,19)			10(8.5,11)		
	3	101	17(15,19)			10(8,11)		
	4	97	17(16,19)			10(9,11)		
	≥5	30	19(15,19)			10.5(7,11)		
ETS training	Yes	193	18(16,19)	Z=-3.602	**0.000**	11(9,11)	Z=-2.801	**0.005**
	No	88	17(13.5,18)			10 (7,10)		

^a^IQR: Interquartile range

^b^Calculated using Mann Whitney U Test

^C^Calculated using Kruskal-Wallis H test

## Discussion

Studies regarding the knowledge and practice of ETS among nurses have been conducted in several countries, as ETS concerns the safety of mechanically ventilated patients [4, 8, 14, 15]. However, little has been revealed from Chinese ICUs in recent years. The results of our study show that Chinese intensive care nurses were aware of the majority of the evidence-based ETS practices and believed their practices followed over half of the guideline items. However, over half of them were unaware of the difference between open and close suctions and the pros and cons of using hyperinflation. Almost 50 % of nurses believed some of their clinical practices did not follow the evidence-based recommendations, such as not routinely using normal saline and using 80–120 mmHg suction pressure during endotracheal suctioning. Receiving ETS training is a significant influencing factor for both the awareness and adherence level of intensive care nurses.

However, there can be no direct comparison between our results and others due to the different tools used. We consider our results to be consistent with those from the studies of Negro et al. [4], Varghese and Moly [25], Heidari and Shahbazi [12], who revealed that over one-third of nurses were unaware of guidelines regarding the tracheal suctioning procedure.

In China, such an awareness level of evidence-based ETS recommendations may be partly due to the inaccessibility of the guidelines to clinical nurses [26]. Even though several English ETS guidelines exit, there was none in Chinese before the adapted guideline by Hu et al. [21]. Many intensive care nurses in Chinese hospitals felt unable to access those English guidelines due to language barriers [26]. In addition, insufficient training experiences may also explain this low to moderate level of awareness and adherence [18]. As shown in our study, nurses who received training demonstrated significantly higher awareness and adherence than those who did not. Nevertheless, one-third of the participants in our study did not receive any ETS-specific training. This is similar to a study by Xu et al. [27] that found that nearly 25 % of intensive care nurses did not receive ICU nursing skills training over 6 months, including those related to ETS. This may be related to the large number of intensive care nurses and lack of systematic training in Hunan Province [17]. In 2018, there were 1847 intensive care nurses in tertiary hospitals along in Hunan [27]. The Specialized ICU Skills Training Center was founded in 2009, but they only conducted 23 systematic trainings that accommodated approximately 1300 nurses up to the end of 2020 [28]. In addition, many hospitals provide one-on-one coaching or a few days of prework training for new nurses entering ICUs, but these are usually not based on up-to-date recommendations for ETS practice and are scarcely considered systematic trainings [27]. Thus, we suggest that (1) more provincial or even nationwide specialized ICU skill training sessions should be conducted to disseminate evidence-based practices, including for ETS, and (2) these hospital- or unit-level coaching and training sessions should incorporate ETS recommendations and target both new and experienced nurses to update their knowledge and skills regarding ETS.

 Our study findings showed that a large proportion of the nurses lacked knowledge of certain aspects of the guidelines. For example, almost two-thirds of intensive care nurses were unaware of the insignificant differences between open and closed suctioning in clinical outcomes (i.e., oxygen saturation, cardiovascular instability, secretion removal, environmental contamination, and cost). This was an unsurprising result, as there have been contradictory findings in the past two decades regarding the comparisons of these two suctioning methods [29, 30]. Some researchers found that the two suctioning methods differed in affecting the heart rate [29, 31], while no difference was found in other investigations [32, 33]. Nevertheless, the methodological flaws of some studies made their research findings less convincing and led to the failure to generate strong recommendations [1, 30]. Recommendations in our study were developed by incorporating the best available evidence [21]. It, therefore, has the potential to be widely applied in clinical ETS practices in China. However, the guidelines were developed in 2017. For further survey and evaluation, the guidelines and survey items need to be updated.

Likewise, over half of the nurses did not know the pros and cons of using hyperinflation (i.e., patients may feel dyspnoeic when the tidal volume is over 900 cc, hyperinflation may relate to increases in mean arterial blood pressure, and using volumes of hyperinflation that are indexed to the size of the patient may assist in minimizing potential difficulties). Elbokhary et al.[34] had similar research findings that nurses retained poor knowledge regarding the adverse effects of hyperinflation. We suggested that ETS training programs should place particular emphasis on low-awareness items to change intensive care nurses’ traditional views towards ETS and promote their acceptance of evidence-based recommendations [25].

Our results revealed that gaps exist between evidence-based ETS practices and current clinical practices [35]. For instance, almost half of the participants believed that their clinical practice differed from or contradicted the evidence-based recommendations, such as not routinely using normal saline and using 80–120 mmHg suction pressure during endotracheal suctioning. To bridge the evidence-practice gap, theoretical education or training alone may not be adequate to influence practice change [10, 12, 36]. Routine training together with individual or group support—such as posttraining follow-up, coaching, the use of support documents such as unit- or hospital-level ETS regulations, web or mobile applications, checklists, reminders, user-friendly pictures, and pocket versions of the guidelines—could potentially elevate the knowledge level and practical ETS skills of intensive care nurses [36–38]. Moreover, leader support alongside guideline implementation is recommended, as leadership has been listed as one of the most important factors influencing knowledge translation in clinical practices [39, 40].

## Strengths and limitations

Few studies have described nursing practices regarding ETS in mainland China. In the present study, we disclose intensive care nurses’ knowledge and practice of ETS in Chinese ICUs and propose recommendations for current clinical nursing practices and training. Limitations existed in this study despite our efforts to minimize the defects during the research process. First, we did not conduct systematic psychometric testing on the questionnaire. Although it was developed based on current ETS recommendations and underwent a brief test-retest reliability and face validity test before the final version, a lack of systematic psychometric testing may limit the comparability of our findings with others. Second, we used a questionnaire survey to investigate ICU nurses’ adherence to ETS recommendations rather than onsite shadowing. There might be a discrepancy between their perceptions and the actual practices of ETS.

## Conclusions

The study findings revealed that Chinese intensive care nurses lacked awareness of several essential evidence-based endotracheal suctioning practices. It also showed that there were considerable gaps between ETS evidence and clinical practices. Further research should emphasize revealing barriers against and facilitators of implementing evidence-based endotracheal suctioning practices and developing context-suitable interventions for guideline implementation. We suggest systematic training on the ETS guidelines along with innovative strategies from implementation science to promote ETS practice changes.

## Data Availability

The datasets used and/or analysed during the current study are available from the corresponding author on reasonable request.

## Abbreviations

AARC: American Association of Respiratory Care
ETS: Endotracheal suctioning
ICU: Intensive care unit
IQR: Interquartile range
SPSS: Software Package Statistical Analysis
RAs: Research assistants
